# The role of the glycome in symbiotic host-microbe interactions

**DOI:** 10.1093/glycob/cwad073

**Published:** 2023-09-23

**Authors:** Rustam Aminov, Leila Aminova

**Affiliations:** The School of Medicine, Medical Sciences and Nutrition, Foresterhill Campus, Aberdeen AB25 2ZD, Scotland, United Kingdom; Midwest Bioprocessing Center, 801 W Main St, Peoria, IL, 61606-1877, United States

**Keywords:** glycome, glycosylation, host-microbe interaction, mucosal surfaces, symbiosis

## Abstract

Glycosylation plays a crucial role in many aspects of cell biology, including cellular and organismal integrity, structure-and-function of many glycosylated molecules in the cell, signal transduction, development, cancer, and in a number of diseases. Besides, at the inter-organismal level of interaction, a variety of glycosylated molecules are involved in the host-microbiota recognition and initiation of downstream signalling cascades depending on the outcomes of the glycome-mediated ascertainment. The role of glycosylation in host-microbe interactions is better elaborated within the context of virulence and pathogenicity in bacterial infection processes but the symbiotic host-microbe relationships also involve substantive glycome-mediated interactions. The works in the latter field have been reviewed to a much lesser extent, and the main aim of this mini-review is to compensate for this deficiency and summarise the role of glycomics in host-microbe symbiotic interactions.

## Introduction

The eukaryotic domain of life appeared much later in evolution than the domains of Bacteria and Archaea. Thus, right from the beginning of the Eukaryota evolution, the organisms of this domain had to embrace the existing and dominant bacterial and archaeal diversity already present in almost all inhabitable ecological compartments of the planet Earth. The emergence of Eukaryotes, therefore, could be considered as a co-evolutionary process because of the competitive pressure of the existing forms of life and inevitable precondition of co-habitation with these life forms in order to survive and replicate. The spectrum of these interactions is extremely broad, including the engulfment of bacterial symbionts in the early evolution of eukaryotes to form organelles such as hydrogenosomes, mitochondria, chloroplasts and possibly other organelles. Thus, the emergence of complex eukaryotes would not be possible without the unique evolutionary path of eukaryotes involving extremely close interactions with the bacterial and possibly archaeal forms of life. This reciprocity still persists ranging from parasitic to symbiotic relationships of eukaryotes with bacteria and archaea.

With the exception of the Mollicutes class of bacteria, which lack cell wall structures, other bacteria possess cell walls that are normally glycosylated. These structures consist of thick peptidoglycan (murein) structures in Gram-positive bacteria, while in Gram-negative bacteria the peptidoglycan layer is thin and the main component of the cell wall is the outer membrane, which includes a large glycolipid complex consisting of lipopolysaccharide (LPS) molecules. In addition to the thick peptidoglycan layer of Gram-positive bacteria, they also express teichoic and lipoteichoic acids (TA and LTA) on their surfaces, which are equivalent to the LPS structures of Gram-negative bacteria. The cell wall glycoconjugate structures of both Gram-positive and Gram-negative bacteria serve as cellular structural components, with the main function of maintaining turgor pressure, and protecting against environmental impacts. Also, these glycosylated molecular patterns of bacterial cell walls are recognised by the corresponding Toll-like receptors (TLRs), which are the part of innate immunity and present in many eukaryotic organisms, including vertebrates and invertebrates. In addition to these glycosylated patterns, the surface of many Gram-positive and Gram-negative bacteria is covered by capsular polysaccharides. There are also a number of other types of glycosylated molecules, in particular glycoproteins. Once thought to be restricted to eukaryotes, post-translational modifications of proteins such as *N*-glycosylation and *O*-glycosylation, are also widespread among the Bacteria and Archaea (Abu-Qarn et al. 2008; Nothaft and Szymanski 2010; Li et al. 2017). The discovery and use of these pathways has opened up new prospects in the field of glycoengineering with the use of bacterial expression systems (Langdon et al. 2009; Solá and Griebenow 2010).

On the eukaryote side, glycosylation is widely represented in many cell types, tissues and organs of eukaryotic organisms. In fungi and invertebrates chitin comprises a main structural component of these organisms which, similarly to the bacterial cell walls, provides the exoskeleton functions and structural integrity. Vertebrates synthesise *N*-linked and *O*-linked glycoproteins, glycolipids, proteoglycans, glycosaminoglycans, glycosylphosphatidylinositol anchors, and free oligosaccharides (Rudd et al. 2022). Glycans play a crucial role in many aspects of eukaryotic cell biology such as development, cancer, infection, and many other diseases. Similarly to other omics technologies, glycomics developments are aimed at rapid, comprehensive and high-throughput characterisation and analyses that are indispensable for the analysis of these highly diverse and dynamic structures (Chen et al. 2021; Trbojević-Akmačić et al. 2022).

Thus, the two main functions of glycans are 1) structural, such as contributing to cell walls, extracellular scaffolds and protein structure-and-function and 2) informational delivery/recognition systems, such as in the recognition of glycans both within the organism and between organisms. In some cases these functions are interchangeable, such as in the case of peptidoglycans and LPS that have evolved as structural components of bacteria but later in evolution eukaryotic hosts started to recognise these glycosylated molecular patterns though a variety of receptors such as TLRs. The plethora of glycan structures on bacterial cell surfaces play an important role in host-microbe interactions. However, while various aspects of glycosylation in virulence and pathogenicity have been studied in great detail during the past two decades (Szymanski and Wren 2005; Poole et al. 2018), much less is known about its role in the mechanisms of symbiotic relationships. Thus, the main aim of this mini-review to overview the emerging field of glycomics in host-commensal/symbiotic interactions. The definition of eukaryotic hosts in this review refers to multicellular eukaryotes.

## Mammalian glycome

Glycosylation of proteins and lipids is involved in numerous functions of mammalian cells including protein folding, trafficking, targeting, recognition, adhesion, recycling, degradation, and other functions. The genes encoding the glycosylation functions comprise a substantive proportion of all protein-encoding genes—between 1% and 2% of eukaryotic genomes (Lairson et al. 2008). Two main protein glycosylation mechanisms are *N*- and *O*-glycosylation. The structure of the former is based on formation of *N*-glycosyl bond between the monosaccharide moiety and an asparagine residue belonging to the consensus sequence Asn-X-Ser/Thr/Cys, with a more frequent presence of Ser and Thr compared to Cys (Toustou et al. 2022). The *O*-glycosylation includes the bond formation through the modified hydroxyl groups of serine or threonine. While the extent and diversity of *N*-glycosylation appears to be more prevalent and involved in a variety of cellular functions, *O*-glycosylation of proteins seems to be biased towards providing resistance and barrier functions (Bergstrom and Xia 2022). The repertoire of genes encoding the glycosylation processes in mammalian cells include more than 700 genes (Nairn et al. 2008). The transcriptional study of this set of glycosylation genes suggested a model, where the glycan abundance largely correlated with the transcriptomic activity of genes encoding biosynthetic and catabolic enzymes. Epigenetic mechanisms are also involved in the diverse glycosylation processes (Indellicato and Trinchera 2021).

## Eukaryotic innate immunity receptors

Eukaryotic hosts possess a number of innate immunity receptors that recognise several conserved molecular patterns, including molecules originating from microorganisms. The repertoire of these receptors includes Toll-like receptors (TLRs), retinoic acid-inducible gene (RIG)-I-like receptors (RLRs), C-type lectin receptors (CLRs), nucleotide oligomerization domain (NOD)-like receptors (NLRs), sialic-acid-binding immunoglobulin-like lectins (Siglecs), and DNA and RNA sensors (Takeda and Akira 2005; Franchi et al. 2009; Barber 2011; Kato et al. 2011; Kumar et al. 2013; Plato et al. 2013). Microbial molecular patterns often include glycosylation and, in the context of infectious agents, the recognition of these glycosylated molecular patterns plays a key role in launching appropriate immune responses (Mahla et al. 2013).

Besides the function of detecting pathogens, these receptors are sensing signals from commensal microbita as well, and these signalling pathways contribute to other aspects of host development and homeostasis. TLRs, for example, were initially discovered for their role in the host development (Anthoney et al. 2018). Signalling through TLR4, which recognises one of the most potent inducers of innate immunity, LPS, suppresses inflammatory reactions in colonic epithelial cells (Narabayashi et al. 2022). The same type of signalling via LPS from the gut commensal *Bacteroides vulgatus*, with a weak agonistic activity, extinguishes intestinal inflammation and restores gut immune homeostasis (Steimle et al. 2019). The collection of commensal LPS may actually silence TLR signalling to induce immune tolerance and promote homeostasis in the gut (d'Hennezel et al. 2017).

Another Toll-like receptor, TLR2, which recognises bacterial glycopeptide structures such as peptidoglycans, lipopeptides and LTA, is also involved in the successful commensal colonisation and the establishment of host-microbe symbiotic relationships (Round et al. 2011). Signalling through this system directs IgA-mediated control of the establishment of healthy gut microbiota (Kubinak et al. 2015). The loss of this signalling system severely restricts the production of specific IgAs that shape the composition of a healthy gut microbiota. Contribution of TLR2 signalling to the healthy gut can be also implemented via the strengthening of the gut epithelial barrier function (Karczewski et al. 2010). These authors established that the scaffold protein zonula occludens (ZO)-1 and transmembrane protein occludin are significantly increased in the vicinity of the tight-junction (TJ) structures as the result of this signalling.

Another mechanism of immunomodulation by glycoproteins from commensal and probiotic bacteria includes their signalling via CLRs. *Lactobacillus rhamnosus* GG, for example, produces SpaCBA pili, which are glycosylated by mannose and fucose residues (Tytgat et al. 2016). These pili glycoproteins are recognised by human dendritic cells (DC) via the C-type lectin receptor DC-SIGN, which is a carbohydrate-dependent pattern recognition receptor. Incubation of immature DCs with purified SpaCBA glycosylated pili results in the induction of different cytokines such as IL-6, IL-10, IL-12p40 and IL-12p35 (Tytgat et al. 2016). This work suggests an important role played by fucosylation and mannosylation of pili of probiotic bacteria in host microbe interaction and immunomodulation.

## Glycosylation of immunoglobins

The role of glycosylation was extensively studied in several classes of immunoglobulins. Alterations in IgG glycosylation, for instance, were associated with different pathologies, although they may happen in the healthy state as well (Klasić and Zoldoš 2021). The patterns of IgG glycosylation are very variable even in the healthy state and depend on genetic background and a number of epigenetic mechanisms. The latter mechanisms, which include DNA methylation and histone modifications, contribute to a variety of alternative IgG glycosylation patterns. Another level of complexity is added by posttranscriptional regulation of IgG glycosylation via small non-coding RNA molecules. Moreover, genome-wide association studies of IgG glycosylation demonstrated the involvement of many transcription factors, receptors, signalling molecules, chromatin remodelers, and other modifiers. In diseases with a pro-inflammatory component such as inflammatory, autoimmune, infectious, cardiometabolic and neoplastic diseases as well as in ageing, the patters of IgG glycosylation in serum show a general trend towards a decreased level of galactosylated and sialylated glycans (Pezer 2021).

IgAs are the most abundant immunoglobulins produced by mammals, which exist in monomeric form in the serum and also in polymeric forms on mucosal surfaces and external secretions (secretory IgA (SIgA)). SIgAs are derived from naïve B cells, which are then recruited into the IgA repertoire upon recirculation in Peyer’s patches (Bunker et al. 2017). These polyreactive IgAs that have innate specificity towards the microbiota are secreted by mucosal surfaces and, besides pathogen clearance, are also involved in the establishment of robust host-microbial symbiosis thus essentially shaping the structure-and-function of host microbiota (Kato et al. 2014; Donaldson et al. 2018; Nakajima et al. 2018; Rollenske et al. 2021). IgA molecules are extensively glycosylated and glycosylation of IgA has a considerable effect on its immune function (Ding et al. 2022). Although aberrant glycosylation of IgA seems to be associated with various pathological states such as autoimmune disease and cancer, the mechanistic explanations for the involvement of particular IgA glycans in disease are still lacking (Hansen et al. 2021). Thus, the diversity of IgA glycosylation patterns is driven by interaction with microbiota and by internal host functions.

Even less evident are the mechanisms of the commensal/symbiotic relationship driven by SIgA, although there are some clues suggesting the host regulates the non-pathogenic microbiota. In particular, the gut IgA may target bacterial protein complexes involved in dietary polysaccharide utilisation and thus control bacterial growth in the intestine (Joglekar et al. 2019). On the microbial side of the interaction, it has been demonstrated that addition of a probiotic *Lacticaseibacillus casei* strain to the diet of mice significantly increases concentration of SIgA in the intestinal lumen (Aindelis et al. 2023). The observation that anaerobic intestinal bacteria are coated by SIgAs has been made a number of years ago (van der Waaij et al. 1996), and the presence of carbohydrate moieties on SIgAs is essential for the interaction of SIgAs with commensal Gram-positive bacteria (Mathias and Corthésy 2011). The mechanistic explanations though of how this bacterial-IgA interaction regulates the composition, metabolic function and stability of gut microbiota still remain elusive.

## Mucosal surfaces

Mucosal surfaces play an important role in protecting the host organisms against pathogen invasion, while suppressing excessive immune activation against commensal microbiota. Mucosal surfaces are covered by glycosylated proteins (mucins), which are secreted by goblet cells and, in the form of secreted and membrane-bound mucins form a gel-like matrix to protect the underlying epithelial cells. The pattern of *O*-linked oligosaccharides in MUC2 is complex and includes more than 100 variants but their repertoire and relative amounts in the sigmoid colon of healthy individuals are relatively constant compared to other mucins (Larsson et al. 2009). The extent of glycosylation of mucins are extremely high, with up to 80% of the total mass consisting of carbohydrate residues (Bansil and Turner 2018). Consequently, the protection of mucosal surfaces is achieved by a layer of extensively *O*-glycosylated proteins, and epithelial glycans constitute a large proportion of the intestinal mucosa providing resistance and tolerance functions (Bergstrom and Xia 2022). Importantly, the mucosal structure appeared to be complex and consists of b1 and b2 layers, which are represented by distinct MUC2 subtypes with unique glycomes. Thus, the complex mucosal glycome serves as the first line of physical barrier against invading pathogenic microbiota, while providing the nutritional ecological niche for commensals (Lee et al. 2013; Raimondi et al. 2021; Wang et al. 2021). If the glycome structure-and-function is compromised, this results in various pathologies such as inflammatory bowel disease (IBD) (Kudelka et al. 2020), cancer (Khosrowabadi et al. 2022), and a range of autoimmune disorders (Indellicato and Trinchera 2021).

Together with sialylation, which is prominent in the distal colon, fucosylation is one of the most abundant types of glycosylation in the mammalian gastrointestinal tract (Pickard and Chervonsky 2015). Its key role in symbiotic host-microbe interaction has been established in many studies, in particular with the species of *Bacteroides*, which induce extensive fucosylation of the host mucin (Fletcher et al. 2009). Induction of fucosylation involves a complex signalling network that requires sensing of TLR agonists and production of IL-23 by dendritic cells, activation of innate lymphoid cells and expression of α1,2-fucosyltransferase-2 by IL-22-stimulated intestine epithelial cells (Pickard and Chervonsky 2015). Moreover, microbiota-induced fucosylation of TLR4 alters its activity and induces non-inflammatory signalling cascade, with the induction of genes important for colonisation, recovery from dysbiosis, and the establishment of fucose-mediated gut homeostasis (Nanthakumar et al. 2023). Besides the use of fucose as a substrate for growth, commensals such as *Bacteroides* incorporate exogenous fucose into their own glycans (Coyne et al. 2005), which contributes to the enhanced fitness of commensals in the gut environment (Fletcher et al. 2009). Alongside with response to, and maintenance of, commensal symbiotic microbiota, fucosylation also plays other important roles contributing to the gut and systemic protection against infection and inflammation (Pickard and Chervonsky 2015). For example, fucosylation that is mediated by epithelial interleukin-22 receptor IL-22RA1 confers colonisation resistance towards the intestinal opportunistic pathogen *Enterococcus faecalis* (Pham et al. 2014). Defects in intestinal fucosylation also result in increased susceptibility to infection by *Salmonella Typhimurium* (Goto et al. 2014). At the same time, the fucosidase activity of the commensal bacterium *Bacteroides fragilis* contributes to the enhanced growth and invasion of *Campylobacter jejuni* (Luijkx et al. 2020).

On the host side, fucosylation may modulate antitumor immunity and, therefore, could be considered as one of the immunotherapeutic approaches in the treatment of cancer (Adhikari et al. 2022). Fucose itself could be also considered as a potential therapeutic molecule in autoimmune diseases with the immune-mediated inflammation such as IgA nephropathy (Qing et al. 2022). L-fucose treatment ameliorates DSS-induced colonic inflammation and intestinal epithelial injury via accelerating the proliferation of intestinal stem cells (Tan et al. 2022). L-fucose supplementation also activates Treg cells thus contributing to the resolution of intestinal inflammation (Feofanova et al. 2022).

Other major glycans in mammals include sialic acids, which are nine-carbon sugars such as *N*-glycolylneuraminic acid (Neu5Gc) and its precursor *N*-acetylneuraminic acid (Neu5Ac) (Jennings et al. 2022). In humans, a mutation in the gene encoding CMP-Neu5Ac hydroxylase makes it inactive so humans cannot convert Neu5Ac to Neu5Gc (Irie et al. 1998; Chou et al. 2002), so only traces of the latter can be detected, presumably from dietary sources. Every cell type in the human body is decorated by Neu5Ac, and these molecules are involved in many functions including cell adhesion, signalling, immunity, brain function, and development. The presence of these glycans on host cell surfaces, for example, is important for self-recognition and thus for the maintenance of homeostatic relationships between the immune system and other cell types in the body. Sialylated host glycans are recognised, for example, by complement Factor H to suppress complement-mediated activation (Blaum et al. 2015) or by CD33-related Siglecs to curb the responses of innate immunity cells (Vitale et al. 1999).

Mucosal surfaces of the mouth, airway, gut and vagina are especially rich in sialoglycans, and this negatively charged mucus layer provides physical protection against bacterial invasion (Lewis and Lewis 2012). During the coevolution of human host and bacterial pathogens, the latter have developed the preference for Neu5Ac, including bacterial adhesins and major toxin classes that recognise Neu5Ac-containing glycans as receptors (Jennings et al. 2022). Besides, bacterial pathogens also synthesise surface structures such as lipooligosaccharide, LPS and polysaccharide capsules to mimic the human host with Neu5Ac in order to evade the detection by the immune system. These strategies could be also implemented by commensal species of bacteria that have dual lifestyles such as bacteria belonging to the *Haemophilus* genus.

In addition to the fucosylated glycans, sialylated glycans are the most abundant on mucosal surfaces, especially in the distal gut. These and other host glycans represent a substantive nutrient source for the gut microbiota (Raba and Luis 2023). Commensal gut bacteria such as strains of *Ruminococcus gnavus* are able to grow on mucin substrates and this property is aided by multiple sialidases encoded by this species (Crost et al. 2016). On a broader scale, a bioinformatic analysis of bacterial genomes from the human gut revealed that 43% of 2,662 genomes from ca. 80 genera encode the genes relevant for Neu5Ac metabolism, such as sialidase or sialic acid catabolism genes (Coker et al. 2021). Many biochemical and structural details of sialic acid utilisation by gut microbiota, including sialidases, transporters, and catabolic enzymes, are now well established (Bell et al. 2023).

At the same time, we cannot declare that our understanding of processes involving sialic acid is comprehensive. The well-characterised sialidases from the human microbiome belong to the GH33 family, but a recent bioinformatic assessment of metagenomes in public databases also revealed a large number of putative GH156 sialidases (Mann et al. 2022). These sequences were almost exclusively associated with human microbiomes and represented by the Bacteroidota, Verrucomicrobiota and Firmicutes_A phyla. Interestingly, a greater variety and abundance of these sialidase genes was observed in the metagenomes from traditional hunter-gatherer or agriculturalist societies compared to industrialised societies, especially if compared to individuals with IBD. These observations suggest that, similar to fucose and fucosylation, the involvement of sialic acid and sialylation in human health can be more complex than simply the physical protection of the host and nutritional source for commensal bacteria, and these properties have to be investigated further (Sokolovskaya et al. 2022).

## Host-microbe interface at mucosal surfaces

Several investigations suggested that the commensal microbiota contributes to the normal structure-and-function of mucosal surfaces. The human isolate of *Bifidobacterium dentium*, for example, binds to the intestinal mucus and upregulates the major mucin MUC2 and modulates goblet cell function via autophagy and calcium signalling pathways (Engevik et al. 2019). There are a significant number of commensal gut bacteria that are able to degrade the host’s mucin and use it as a nutrient source (Glover et al. 2022). These bacteria induce low-level inflammation in the gut but also improve epithelial TJ barrier function and protect against excessive inflammation incited by pathogenic bacteria (Pan et al. 2022). In particular, the authors established that gut commensal bacteria may enhance the epithelial TJ barrier function by regulating transcription of TJ protein genes such as ZO-1, occludin, claudin-1 and E-cadherin,

The ability to use the host polysaccharides is also an important colonisation factor for symbionts. The presence of polysaccharide utilisation loci in symbionts is indispensable for successful colonisation and persistence, which is provided by close association of commensal bacteria with the host through penetrating the colonic mucus and residing deep within crypt channels thus providing resilient gut colonisation (Lee et al. 2013). The breakdown of mucin requires several enzymatic steps. Some members of the human gut microbiota express endo-acting *O*-glycanases to initiate mucin degradation (Crouch et al. 2020). Another enzyme participating in mucin degradation, *O*-glycopeptidase, was identified in *Akkermansia muciniphila* and extensively characterised (Trastoy et al. 2020; Medley et al. 2022). This enzyme possesses a metzincin metalloprotease catalytic motif but the active site specifically recognises a *N*-acetylgalactosamine (GalNAc) residue α-linked to a serine or threonine residue. Also, the enzyme has a carbohydrate-binding module, which may be involved in the recognition of substrates that include a carbohydrate component. Due to its beneficial properties, this bacterium is proposed as one of the next-generation beneficial microorganisms (Cani et al. 2022). At the same time, the situation with mycolytic bacteria could be a double-edged sword since, for example, the presence of *A. muciniphila* under dietary nutrient restriction may contribute to the proliferation of pathobionts in the gut (Sugihara et al. 2022).

Similar contradictory effects concerning the use of host glycopeptides have been observed with other commensal and pathogenic gut bacteria. The commensal gut bacterium *Bacteroides thetaiotaomicron*, for example, regulates the production of fucosylated glycans by intestinal enterocytes to ensure that the nutritional requirements of the bacterium are met in the competitive gut environment (Hooper et al. 1999). The fucose-sensing system of the gastrointestinal pathogen enterohaemorrhagic *Escherichia coli* (EHEC) senses fucose and controls expression of virulence and metabolic genes (Pacheco et al. 2012). Thus, the cleavage of fucose from mucin by *B. thetaiotaomicron* contributes to the virulence of EHEC, since sensing fucose activates the FusKR signalling cascade, which modulates virulence gene expression. Other responses of gut commensal bacteria in contact with the mucosa and mucin include the induction of a number of genes involved in membrane transport, chemotaxis, motility, and horizontal gene transfer (Patterson et al. 2017), but the molecular mechanisms involved in up-regulation of these genes are presently unknown.

The general *O*-glycosylation system is an important part of physiology of intestinal symbionts such as the species of *Bacteroides*, which contributes to their numerical prevalence in the human gut (Fletcher et al. 2009). In particular, the species of this genus are capable of using glycosylation pathways similar to host pathways for fucosylation of their surface capsular polysaccharides and glycoproteins (Coyne et al. 2005). The fucosylated surfaces are expressed simultaneously by host and symbiont, and the lack of this ability by the symbiont results in colonisation deficiency. On the host’s side, temporary anorexia caused by infections could also result in fucosylation deficiency but, surprisingly, the host allocates its own resources for rapid fucosylation of the intestinal epithelium to maintain symbiotic host-microbe interactions during sickness (Pickard et al. 2014). The ability of certain pathogens such as *Klebsiella pneumoniae* to metabolise fucose helps them to exploit the host’s fucosylation system and promote their own gastrointestinal colonisation (Hudson et al. 2022). Interestingly, some bacteria that are usually considered as environmental, such as *Bacillus subtilis*, also possess affinity and bind to fucosylated glycans (Tiralongo et al. 2018).

## Glycosylation in bacteria

Glycosylation of bacterial surface structures serve as a protective structures against environmental impact. In regards to interaction with eukaryotic organisms, structures such as LPS from pathogenic bacteria possess very potent immunomodulating ability. The role of bacterial glycosylation in virulence and pathogenicity have been intensively characterised during the past two decades. Several mechanisms of glycosylation that contribute to bacterial virulence and facilitate several stages of infection were identified (Szymanski and Wren 2005; Ribet and Cossart 2010). A large number of glycosyltransferases and the corresponding glycoproteins that are important for virulence have been identified in pathogenic bacteria (Lu et al. 2015). Besides, bacterial pathogens employ the strategy of interference with the host signalling systems via the delivery of bacterial effectors into host cells to modify host targets for successful infection (Mak and Thurston 2021). Some of these effectors possess glycosyltransferase activities.

Progress on understanding the role of the bacterial glycome in symbiotic relationships with the host has been much more modest. Only a limited number of examples covering the symbiotic aspect of glycosylation can be found in the published literature. Certain glycosylation patterns, however, are shared by pathobionts and symbionts. In pathobionts such as *Streptococcus pneumoniae* and *S. agalactiae*, for example, surface glycoproteins serve as adhesins that allow adherence to mucosal surfaces, invasion of underlying tissues and subsequent transition to disease (Chan et al. 2020). In symbiotic bacteria, surface glycoproteins are indispensable for beneficial interaction with the host, without tissue invasion and disease.

Other functional roles of glycosylation in bacteria and archaea include the interaction with phages and serving as an important component in structure-and-function of biofilms. Glycosylated surfaces of bacteria such as LPS in Gram-negative bacteria or peptidoglycan and teichoic acids in Gram-positive bacteria serve as specific host receptors for the attachment, for example, of tailed bacteriophages on bacterial host surfaces to initiate the first step of infection (Nobrega et al. 2018). At the same time, bacteria can defend themselves against bacteriophages by masking potential phage binding sites via target glycosylation (Harvey et al. 2018). Thus, the bacterial and archaeal glycosylation patterns as a mode of recognition/avoidance appeared well before the emergence of eukaryotes and the corresponding host-microbe interactions via the glycome.

The current estimates suggest that 40%–80% of all bacteria and archaea on Earth reside within the biofilm communities (Flemming and Wuertz 2019). As a main mode of existence in the microbial world, biofilms possess a larger range of functionalities compared to the planktonic state cells, including some aspects of multicellularity (Penesyan et al. 2021). The role of microbial biofilms in disease is well documented as well as their role in beneficial host-microbe interaction (de Vos 2015). Bacterial and archaeal cells within biofilms are embedded within an extracellular matrix, which is mainly composed of extracellular polysaccharides, with the inclusion of nucleic acids, proteins, and lipids. The role of polysaccharides in the glycome-mediated host-microbe interaction is discussed in the next section.

## Capsular polysaccharides of bacteria

Another mechanism of symbiotic host-microbe interaction is implemented via the bacterial surface structures such as capsular polysaccharides (CPs). These are known virulence factors but in bacterial symbionts they may play a different role. It has been revealed in several investigations that the presence of multiple capsular polysaccharides is essential for bacterial colonisation in host-microbe symbiosis (Coyne et al. 2008; Liu et al. 2008). If a symbiont lacks the surface molecules that are normally recognised by the host and therefore identified as a “friendly” bacterium, the host responds by its sequestration in luminal casts as a potentially harmful microbe to avoid dysregulated T cell activation (Sassone-Corsi et al. 2022).

Also, it has been established in the early years of studies of CPs from commensal bacteria such as polysaccharide A (PSA) from *B. fragilis* that these molecules have potent immunomodulatory activities and may prevent intestinal inflammatory disease (Mazmanian et al. 2008). The mechanism of PSA action is complex and the host responses include the combination of pro-inflammatory cytokines and anti-inflammatory surface receptors, which suggest the potentials for both outcomes, with promotion and inhibition of inflammatory disease (Alvarez et al. 2020). Still, in further studies of PSA effects on antigen presenting cells a significant polarisation towards anti-inflammatory macrophages has been demonstrated (Zhou et al. 2022). Moreover, inactivation of PSA reduces the induction of host anti-inflammatory factors (Arnolds et al. 2023). On a broader scale, a bioinformatic inspection of publicly available genomic sequences for the presence of PSA homologues revealed its wide occurrence in taxonomically diverse bacteria that included Bacteroidales, Erysipelotrichales, Clostridiales, and Bacillales orders (Neff et al. 2016). Experimental verifications with some of these bacteria confirmed that PSA-synthesising bacteria significantly increase the production of Tregs and IL-10 and also attenuate experimental colitis in mice. Other bacterial polysaccharides also display an encouraging therapeutic potential (Khan et al. 2022).

## Glycosylated serine rich repeat proteins in symbiotic bacteria

Glycosylated serine rich repeat proteins (SRRPs) of Gram-positive bacteria represent a family of adhesins that are involved in attachment of these bacteria to various host and bacterial structures (Lizcano et al. 2012). These are different from other bacterial adhesins such as lectins, which form elongated multi-subunit protein structures, fimbriae and pili, with no apparent role of glycosylation in binding to the host cells. As the name suggests, SRRPs contain domains with alternating serine and threonine residues, which are *O*-glycosylated. Initially, it was thought that SRRPs are involved exclusively in pathogenic processes to provide the first steps in colonisation through providing close contact with the host cells, which then can be followed by bacterial invasion and disease (Chan et al. 2020). A recent bioinformatic analysis, however, has revealed the presence of the non-canonical accessory secretion system in the genomes of gut commensal bacteria, which are associated with SRRPs (Latousakis et al. 2020).

While this type of glycosylation is relevant to pathogenic bacteria, a number of recent investigations discovered the presence of SRRPs in a variety of non-pathogenic microbiota, including bacteria that are commensals such as *Streptococcus salivarius* (Couvigny et al. 2017) or the generally recognised as safe (GRAS) bacteria such as a probiotic bacterium *Limosilactobacillus reuteri* (formerly *Lactobacillus reuteri*) (Sequeira et al. 2018). In *S. salivarius*, the presence of glycosylated SRRPs is pivotal for adhesion and colonisation of mucosal surfaces by this commensal bacterium. In *L. reuteri* strains isolated from rodent and pig hosts, the patterns of SRRP glycosylation appeared to be different (Latousakis et al. 2019). This suggests that different SRRP glycosylation patterns in *L. reuteri* strains isolated from different hosts are presumably host-specific and reflect the coevolutionary trajectory of this host-microbe interaction.

Recognition of a specific host glycan repertoire may determine a pathogenic or commensal potential of a bacterium. These recognition mechanisms were investigated using the Siglec-like binding regions (SLBRs), which are found within the context of SRRPs in streptococci (Bensing et al. 2022). The authors employed five representative SLBRs and their variants to identify the regions of host glycan receptor binding sites. They concluded that the conserved sialic acid-recognition motif governs general specificity while sequence diversity in surrounding loop regions allows the SLBR to select between related sialoglycans. Binding of the gut symbiont *R. gnavus* to intestinal mucus is also mediated by sialic acid (Owen et al. 2017). This bacterium is capable of degrading mucins via an intramolecular trans-sialidase, which consists of a catalytic domain and a sialic acid-binding carbohydrate-binding module. The latter module is responsible for mucin binding and this binding module seems widespread among the Firmicutes (Bacillota).

## Conclusions

Understanding the mechanisms of host-microbe interaction implemented via the corresponding glycomes has substantially improved during the past several years. The progress, however, has been mainly focused on virulence and pathogenic aspects of this interaction. The majority of microbiota though do not cause disease and even could be symbiotic and mutually beneficial. Nonetheless, this aspect of host-microbe interaction has received much less attention, in particular in revealing the role played by the respective glycomes in symbiotic interactions. At the same time, some mechanisms of host-microbe interaction are shared within the pathogen-commensal-symbiont continuum. Close proximity is required for any type of interaction and many bacteria share common mechanisms such as adhesion to host tissues to provide this functionality, irrespective of their pathogenic potential. Glycosylated SRRPs that are involved in adhesion to host or bacterial surfaces, for example, are present in both pathogens and commensals. Similarly, the use of host glycopeptides such as cleaved mucins and glycoproteins on epithelial surfaces by commensal microbiota helps to maintain diversity and stability. However, the use of host glycopeptides by commensal bacteria also contributes to proliferation and virulence of pathobiota. Thus, there is a very delicate balance between the maintenance of commensal microbiota and potential overgrowth of pathogens/pathobionts, which is mediated by the rate, diversity and architecture of glycopeptides and lectins produced by the host.

Much less is known about the role of glycosylated SIgA in the host-microbe symbiotic relationship. Bacteria in the intestine are coated with SIgA and glycosylation of SIgA is crucial for this process. It is not clear, however, through what mechanism this bacterial-SIgA interaction may regulate the intestinal microbiota. One of the ways could be SIgA targeting bacterial protein complexes that are involved in the utilisation of dietary polysaccharides thus restricting substrate availability and bacterial growth (Joglekar et al. 2019). Successful gut colonisation by commensals could be also aided by adhesion of surface capsular polysaccharides to the intestinal epithelium, with the involvement of SIgA (Donaldson et al. 2018). Depending on the nutritional status, intestinal bacteria may modulate binding of SIgA due to the loss of glycan-mediated interactions between bacteria and antibodies (Huus et al. 2020). Also, bacteria in the gut produce short chain fatty acids and the effect of one of them, acetate, appeared to be differentially regulating IgA reactivity to commensal bacteria (Takeuchi et al. 2021). Thus, the regulatory mechanisms implemented at the SIgA level are much more complex and involve several different mechanisms, which is not surprising given the high specificity of immunoglobulins produced and the extreme diversity of microbiota and the corresponding antigens present in the gut.

Glycosylated molecules on the surface of Gram-negative bacteria such as LPS are known as one of the most potent immunomodulatory molecules that are recognised by the TLR4/myeloid differentiation factor 2 (MD-2) receptor complex, followed by the signalling pathway activating NF-κB, with a vigorous induction of pro-inflammatory immune responses. LPS from commensal bacteria such as *B. vulgatus*, however, display only weak agonistic activity (Steimle et al. 2019). Moreover, in contrast to the strong pro-inflammatory responses induced by LPS of pathogenic bacteria, this weak agonistic activity results in attenuation of inflammatory responses and restoration of intestinal immune homeostasis. On a broader scale, the total gut microbiome LPS, which is primarily derived from Bacteroidales, displays overall immunoinhibitory properties via silencing of TLR signalling (d'Hennezel et al. 2017). The authors suggested that underacylated lipid A structures in LPS of commensal bacteria may be responsible for the inhibition of this pro-inflammatory signalling. This anti-inflammatory activity of LPS from gut commensals could be potentially used for therapeutic purposes to treat diseases associated with chronic inflammation such as IBD (Lin et al. 2020).

A similar situation may exist with another major glycopeptide, that is the peptidoglycan of Gram-positive bacteria. The cell wall of *Staphylococcus aureus* contains peptidoglycan-embedded TLR2 ligands, which may either induce pro-inflammatory responses or act as anti-inflammatory modulators to curtail the pathogenic potential of this bacterium and its toxins (Mele and Madrenas 2010). It has been demonstrated recently that the cell wall glycoconjugates of the Firmicutes (Bacillota) enter the systemic circulation of the host and induce the secretion of cytokine IL-34, which stimulate macrophages and thus provides a protection against bacterial infectious agents (Jordan et al. 2023). To prevent the excessive accumulation of bacterial glycoconjugates that may result in immunopathology, IL-34-mediated mTORC1 activation in macrophages eliminates polymeric glycoconjugates, while smaller glycoconjugates are sequestered by albumin. This work demonstrates the delicate balance of host-microbe interactions implemented via the glycoconjugates of symbiotic Firmicutes bacteria, which provides protection of the host against infections and, at the same time, limits excessive inflammatory responses that could be detrimental to the host.

Another example of differential host responses to TLR ligands of pathogenic and commensal bacteria is sensing bacterial flagellins by TLR5, which triggers a rapid pro-inflammatory host response in the case of pathogens (Ramos et al. 2004). The crucial step in the biosynthesis of bacterial flagellins is glycosylation (Logan 2006), and the lack of flagellar glycosylation in pathogens such as *C. jejuni* and *Helicobacter pylori* results in lack of flagellar biosynthesis, with the loss of motility and diminished virulence (Yakovlieva et al. 2021). The glycosylation pattern of flagellin in *C. jejuni*, however, does not affect TLR5 signalling, the reduced signalling is due to the primary protein structure (de Zoete et al. 2010). At the same time, mutations that weaken the TLR5 signalling by *C. jejuni* are compensated for by extensive interactions between the outer domains of the flagellin subunits through the glycosylation of key residues (Kreutzberger et al. 2020). Thus, the reduced TLR5 signalling through the changes in the primary protein structure are still compensated with glycosylation to retain the primary function of the *C. jejuni* flagellin, which is motility. In another pathogen, *Burkholderia cenocepacia*, the primary mechanism of avoiding host immunity is glycosylation of flagellin that reduces its recognition by the host TLR5, thus allowing it to evade immune surveillance to execute a successful infection (Hanuszkiewicz et al. 2014). Thus, flagellin glycosylation in pathogens may play an important role in evading immune surveillance and in sustaining the primary motility function to compensate for losses associated with the former. Flagellated representatives of the commensal microbiota also have to limit a potentially excessive inflammatory response due to TLR5 signalling to ensure stable colonisation and homeostatic conditions. Flagellins of commensal bacteria such as *Roseburia hominis*, for example, display much lower activation of pro-inflammatory responses, while promoting and regulating innate immunity (Patterson et al. 2017). Presently, it is not clear, however, whether the reduced TLR5 signalling strategy of commensal microbiota is due to the selection of specific primary structures of flagellin or selection of specific glycosylation patterns. There are some indications that immunological attenuation of flagellins in certain commensal microbiota is most likely achieved through specific amino acid substitutions in flagellin rather than its glycosylation (Kajikawa et al. 2022). More research is needed, however, to reveal the entire range of mechanisms that may include the glycome interaction between the host and commensal microbiota.

The long-term co-evolution of eukaryotic hosts and bacteria resulted in the survival and replication strategies that culminated in the glycome-mediated interface for their interactions. For pathogenic bacteria, glycosylation served as an important virulence and pathogenicity mechanism to ensure a successful infectious process. Another host-microbe interaction archetype exploited a mutual cohabitation model to result in a strategy that uses the glycome interface for symbiotic relationships (Table 1). The traits that have been selected during the co-evolutionary process allow the symbionts the glycan-mediated recognition of the host involving a number of interactions such as finding the right host, attachment, colonisation, foraging the host’s nutrients, and modulation of host immunity.

**Table 1:** The glycome-mediated interface of host-microbe symbiotic interactions.

HOST^a^	MICROBIOTA
PAMPs DAMPs	LPS, LOS, Murein, TA, LTA, CPs, SRRPs and other glycome components of pathogens
SyAMPs SAMPs	LPS, LOS, Murein, TA, LTA, CPs, SRRPs and other glycome components of symbionts
Secretion of mucin and SIgA Signalling/response molecules Innate/acquired immunity	Protection against pathogens and econiche for symbionts. Signalling by symbionts provides proper host development and stable homeostatic conditions.

^a^The host glycome is shown in the left column and that of the microbiota is shown in the right column. Sensing of glycosylated patterns by the host include Pathogen-Associated Molecular Patterns—PAMPs, Danger-Associated Molecular Patterns—DAMPs, Self-Associated Molecular Patterns—SAMPs, and Symbiont-Associated Molecular Patterns—SyAMPs, which then trigger the corresponding signals and responses. Glycosylated proteins of host mucosal surfaces such as mucin provide physical protection and provide nutrients for symbionts. Secreted glycosylated molecules such as SIgA contribute to the establishment of symbiotic microbiota, while limiting colonisation by pathogens. In pathogens, glycoconjugates comprising the cell wall, cell surface structures such as SRRPs and CPs serve as virulence factors, while in symbionts they allow the evasion of immune surveillance, attachment, colonisation, and proliferation. These molecules signal to the host immune system and provide colonisation resistance against pathogens. The induction and use of host glycans by symbionts contributes to homeostasis.

In eukaryotic organisms, the evolution of protective mechanisms against foreign invaders and their own cells that are damaged or dysregulated, including cancer cells, has led to the emergence of the adaptive immune system. This evolution, however, brought up a conundrum of how to differentiate between the molecular patterns of self and non-self, the latter including the patterns of uncontrolled proliferation or cell damage. One of the ingenious solutions to this dilemma became the recruitment of specific glycans providing the recognition patterns of self to prevent self-destruction (Table 1). In response to this acquisition, however, both the pathogenic and symbiotic microbiota have evolved to use the glycan patterns that imitate the host’s patterns in order to avoid host immune system surveillance. The resulting selective pressure, imposed by pathogenic microorganisms, contributed to host glycan diversity and to the maintenance of a high-level of glycan polymorphism as well as production of antibodies against foreign glycans (Gagneux et al. 2022). Such glycan polymorphism represents a great challenge to symbiotic microbiota in terms of optimal adaptation to their hosts.

Another problematic aspect of the glycome-mediated relationship between the host and commensal microbiota is in the rapid change of the latter due to our modern lifestyle and dietary habits. While the host-microbiota symbiotic relationships have been evolving for millions of years, two major events in human history strongly affected the microbial part of the host-microbe interaction. The first was the advent of early agricultural societies during the Neolithic period about 12,000 years ago and the second, with the start of the Industrial Revolution about 300 hundred years ago. Both have resulted in substantial dietary and lifestyle changes that affected the human microbiome and, therefore, the host-microbe glycome interface. While some of these mutually beneficial interactions may have survived, there are some indications that in modern societies some components of this interaction such as the diversity and abundance of specific sialidases may have diminished, which also correlates with the incidence of IBD (Mann et al. 2022). In general, the gut microbiomes in industrialised societies display diminished diversity and are enriched in oxidative stress genes, possibly reflecting the inflammatory processes common for the contemporary dietary and lifestyle habits (Carter et al. 2023). Thus, the host-microbe interaction that includes the maintenance of symbiotic microbiota via the glycome-mediated immune selection and feeding as well as commensal bacteria contributing to the host’s well-being through the development and maintenance of the immune system and other functions may be seriously compromised. Mechanistic details of this intricate symbiotic relationship, however, are still scarce and more work is needed to uncover the mechanisms that may have a substantive translational medicine impact for the improvement of human health.

##  

*Conflict of interest statement*: None declared.

## Supplementary Material

Glycobiology_CONFLICT_OF_INTEREST_FORM_cwad073
