# Supplementary material for: The role of the glycome in symbiotic host-microbe interactions
Source: Glycobiology. 2023 Sep 23;33(12):1106–16. doi: 10.1093/glycob/cwad073 (PMC10876039; doi:10.1093/glycob/cwad073)
Supplement: Glycobiology_CONFLICT_OF_INTEREST_FORM_cwad073 [file glycobiology_conflict_of_interest_form_cwad073.pdf]

***Glycobiology* CONFLICT OF INTEREST FORM**

Title: The role of glycome in symbiotic host-microbe interaction

Author(s): Rustam Aminov, Leila Aminova

Ms number:

MANUSCRIPT AUTHORS: *Glycobiology* policy requires that, at the time of submission, each author reveal any financial interests or connections, direct or indirect, or other situations *that might raise the question of bias* in the work reported or the conclusions, implications, or opinions stated - including pertinent commercial or other sources of funding for the individual author(s) or for the associated department(s) or organization(s), personal relationships, or direct academic competition. *If* you have been asked to do so by the corresponding author, please complete **Part I** or **Part II**.

When considering whether you should declare a conflicting interest or connection please consider the conflict of interest test: Is there any arrangement that would compromise the perception of your impartiality of that of your co-authors if it was to emerge after publication that you had not declared it?

PLEASE NOTE: The *corresponding* author is required to confirm whether any co-author has a conflict of interest to declare. If the corresponding author is unable to confirm this on behalf of each co-author, the co-author in question is required to complete this Conflict of Interest form email it to the Editorial Office at glycobio2@jhmi.edu. It is the corresponding author's responsibility to ensure that all authors adhere to this policy.

If the manuscript is published, this information will be communicated in a statement in the published paper.

Part I. There have been no involvements that might raise the question of bias in the work reported or in the conclusions, implications, or opinions stated.

Printed name: Rustam Aminov

Signature: R.I. Aminov ____________________ Date: 16/01/2023

OR

Part II. Conflict of interest statement

Sample statement: I hold stock* in [business name], the makers of [product], and am currently conducting research sponsored by this company. I am also a paid consultant for [business name].

* Please declare stock where this reveals a pertinent conflict of interest. Details (number, value) are not required.

My statement is as follows:

Printed name:

Signature: ____________________ Date:
